# In Vitro Interaction between Isavuconazole and Tacrolimus, Cyclosporin A, or Sirolimus against *Aspergillus* Species

**DOI:** 10.3390/jof6030103

**Published:** 2020-07-08

**Authors:** Patrick Schwarz, Eric Dannaoui

**Affiliations:** 1 Department of Internal Medicine, Respiratory and Critical Care Medicine, University Hospital Marburg, D-35043 Marburg, Germany; 2 Center for Invasive Mycoses and Antifungals, Philipps University Marburg, D-35037 Marburg, Germany; 3 Dynamyc Research Group (EA 7380), Faculté de Médecine de Créteil, Université Paris-Est-Créteil-Val-de-Marne, F-94010 Créteil, France; eric.dannaoui@aphp.fr; 4 Unité de Parasitologie-Mycologie, Hôpital Européen Georges-Pompidou, F-75015 Paris, France; 5 Faculté de Médecine, Université de Paris, 75006 Paris, France

**Keywords:** antifungal combination, in vitro, aspergillosis, *Aspergillus*, isavuconazole, immunosuppressor, EUCAST

## Abstract

The interaction of isavuconazole with immunosuppressors (tacrolimus, cyclosporin A, or sirolimus) against 30 *Aspergillus* isolates belonging to the most common species responsible for invasive aspergillosis in humans (*Aspergillus flavus*, *Aspergillus fumigatus*, *Aspergillus nidulans*, *Aspergillus niger*, and *Aspergillus terreus*) was evaluated in vitro by a microdilution checkerboard technique based on the EUCAST reference method for antifungal susceptibility testing. The interpretation of the results was performed based on the fractional inhibitory concentration index. The combination of isavuconazole with tacrolimus, cyclosporin A, or sirolimus, was synergistic for 56, 20, or 10% of the isolates, respectively. Interestingly synergy of the combination of isavuconazole with tacrolimus was also achieved for the majority of azole-resistant isolates of *A. fumigatus*, and for all *A. niger* isolates with isavuconazole minimal inhibitory concentrations ≥ 8 µg/mL. Antagonistic interactions were never observed for any combination tested.

## 1. Introduction

Invasive aspergillosis is a devastating disease in immunocompromised patients associated with a high mortality rate of about 35% [1]. It mostly affects patients with hematological malignancies, especially those with severe and prolonged neutropenia [2], but is also encountered in solid organ transplant recipients [3,4,5]. Voriconazole has long been the treatment of choice for invasive aspergillosis [6], and recently isavuconazole expanded the portfolio of first-line treatments [7], but azole-resistance is increasingly reported in *Aspergillus fumigatus* [8]. In a prospective multicenter international surveillance study, a total of 3788 *Aspergillus* isolates were screened in 22 centers from 19 countries. Azole-resistant *A. fumigatus* isolates were found in 3.2% of the cases [9]. The majority of azole-resistant *A. fumigatus* isolates are resistant due to the TR_34_/L98H mutation in the *cyp51A* gene [10]. The mutation TR_34_/L98H is also found in *A. fumigatus* isolates cultured from soil and compost. These isolates are cross resistant to azole fungicides and genetically related to clinical azole-resistant aspergilli, showing that the fungicides used for the protection of crops and other plants contribute to the emergence of azole-resistance in *A. fumigatus* [11]. The high mortality rate among patients with invasive aspergillosis due to multiple triazole resistant *A. fumigatus* isolates, and the possibility of the worldwide spread of these resistant isolates by the use of fungicides in agriculture, make azole-resistance in *A. fumigatus* a major health problem [12]. Isavuconazole is a new board-spectrum azole antifungal drug with excellent activity against most *Aspergillus* species [13]. Isavuconazole or voriconazole are currently recommended as first-line therapies for pulmonary aspergillosis in Europe [14]. It has been shown that isavuconazole-resistant *Aspergillus* isolates can be cross-resistant to voriconazole [15,16]. Therefore, combination with antifungals or non-antifungal drugs may be interesting to overcome this resistance. Calcineurin inhibitors (e.g., tacrolimus and cyclosporin A) or inhibitors of the mTOR pathway (e.g., sirolimus) are anti-rejection drugs widely used in organ transplant patients [17], and to prevent graft-versus-host disease in allogeneic stem cell recipients [18], but these immunosuppressive drugs also possess intrinsic antifungal activity against selected fungi, including *Candida albicans* [19], *Cryptococcus neoformans* [19,20], *A. fumigatus* [21], *Rhizopus arrhizus* [22], and *Coccidioides immitis* [23]. Calcineurin inhibitors have even exhibited synergy in combination with amphotericin B, posaconazole or isavuconazole against Mucorales [24,25]. Therefore, it is not only of clinical interest, not only if the synergy can also be achieved for *Aspergillus* species, but also to evaluate calcineurin inhibitors as a potential new antifungal class.

Invasive aspergillosis is a devastating disease in immunocompromised patients associated with a high mortality rate of about 35% [1]. It mostly affects patients with hematological malignancies, especially those with severe and prolonged neutropenia [2], but is also encountered in solid organ transplant recipients [3,4,5]. Voriconazole has long been the treatment of choice for invasive aspergillosis [6], and recently isavuconazole expanded the portfolio of first-line treatments [7], but azole-resistance is increasingly reported in *Aspergillus fumigatus* [8]. In a prospective multicenter international surveillance study, a total of 3788 *Aspergillus* isolates were screened in 22 centers from 19 countries. Azole-resistant *A. fumigatus* isolates were found in 3.2% of the cases [9]. The majority of azole-resistant *A. fumigatus* isolates are resistant due to the TR_34_/L98H mutation in the *cyp51A* gene [10]. The mutation TR_34_/L98H is also found in *A. fumigatus* isolates cultured from soil and compost. These isolates are cross resistant to azole fungicides and genetically related to clinical azole-resistant aspergilli, showing that the fungicides used for the protection of crops and other plants contribute to the emergence of azole-resistance in *A. fumigatus* [11]. The high mortality rate among patients with invasive aspergillosis due to multiple triazole resistant *A. fumigatus* isolates and the possibility of the worldwide spread of these resistant isolates by the use of fungicides in agriculture make azole-resistance in *A. fumigatus* a major health problem [12]. Isavuconazole is a new board-spectrum azole antifungal drug with excellent activity against most *Aspergillus* species [13]. Isavuconazole or voriconazole are currently recommended as first-line therapies for pulmonary aspergillosis in Europe [14]. It has been shown that isavuconazole-resistant *Aspergillus* isolates can be cross-resistant to voriconazole [15,16]. Therefore, combination with antifungals or non-antifungal drugs may be interesting to overcome this resistance. Calcineurin inhibitors (e.g., tacrolimus and cyclosporin A) or inhibitors of the mTOR pathway (e.g., sirolimus) are anti-rejection drugs widely used in organ transplant patients [17] and to prevent graft-versus-host disease in allogeneic stem cell recipients [18], but these immunosuppressive drugs also possess intrinsic antifungal activity against selected fungi, including *Candida albicans* [19], *Cryptococcus neoformans* [19,20], *A. fumigatus* [21], *Rhizopus arrhizus* [22], and *Coccidioides immitis* [23]. Calcineurin inhibitors have even exhibited synergy in combination with amphotericin B, posaconazole, or isavuconazole against Mucorales [24,25]. Therefore, it is not only of clinical interest, not only if the synergy can also be achieved for *Aspergillus* species but also to evaluate calcineurin inhibitors as a potential new antifungal class.

## 2. Materials and Methods

### 2.1. Isolates

A panel of 30 clinical *Aspergillus* isolates, from the collection of the parasitology/mycology unit of Hôpital Européen Georges-Pompidou (HEGP), belonging to 5 species responsible for human invasive aspergillosis was used for the experiments (5 *Aspergillus flavus*, 10 *A. fumigatus,* 5 *Aspergillus nidulans*, 5 *Aspergillus niger*, and 5 *Aspergillus terreus*). The isolates of *A. fumigatus* included 5 azole resistant strains (four with TR34/L98H alterations (HEGP-5780, HEGP-4083, HEGP-2659, and HEGP-2664) and one with a G54W mutation (HEGP-4020)). For the other species, isolates were randomly selected to be representative of the species and none of the isolates were known to have specific mechanisms of antifungal resistance. Isolates were subcultured from frozen stocks on Sabouraud dextrose agar slants (Bio-Rad, Feldkirchen, Germany) for 7 days at 35 °C to ensure purity and viability. The reference strains *Candida krusei* ATCC 6258 and *Candida parapsilosis* ATCC 22019 were included in each series of experiments as quality controls.

### 2.2. Medium Preparation

Roswell Park Memorial Institute 1640 (RPMI) medium (with L-glutamine, and pH indicator, but without bicarbonate) (Merck, Darmstadt, Germany) supplemented with dextrose to a final concentration of 2%, buffered with MOPS (Merck) at a final concentration of 0.165 mol/L, and adjusted to pH 7.0 with 1 M sodium hydroxide was used as a test medium. The medium was prepared at double strength to allow a two-fold dilution. After preparation, the medium was sterilized by vacuum filtration through a 0.22 µm pore size filter (Merck).

### 2.3. Drugs and Microplate Preparation

Drug combinations were tested using the EUCAST guidelines for the antifungal susceptibility testing of molds with modifications for a broth microdilution checkerboard procedure, using Nunclon™ delta surface 96-wells microtiter plates for adherent cells (Thermo Fisher Scientific, Darmstadt, Germany). The included drugs were isavuconazole (Pfizer, Berlin, Germany), tacrolimus (Selleck Chemicals, Munich, Germany), cyclosporin A (Selleck), and sirolimus (Selleck). Stock solutions of drugs were prepared in DMSO. Drug dilutions were performed to four times the final concentrations in double strength RPMI medium. All the combinations were studied on a two-dimensional checkerboard with two-fold dilutions. The final concentrations for isavuconazole were 0.03 to 16 µg/mL. The final concentrations for the immunosuppressors were 0.125 to 8 µg/mL. Fifty microliters of each concentration were distributed from Rows 1 to 8 for isavuconazole and from Columns 1 to 11 for the immunosuppressive agents. Column 12 was used a as growth control and contained 100 µL of double strength RMPI medium with DMSO.

### 2.4. Inoculum Preparation and Inoculation of Microplates

Before inoculum preparation, isolates were subcultured a second time on Sabouraud dextrose agar slants and incubated at 35 °C under 95% humidity for 7 days. Spores were transferred to a sterile tube containing water supplemented with 0.1% of Tween 80 by using a wet cotton swab immersed in sterile water. The suspension was counted in a hemocytometer and adjusted to 2 × 10^5^ conidia/mL with sterile water containing 0.1% of Tween 80 in order to prevent the growth of fungi on the surfaces inside the wells [26]. One hundred microliters of the final inoculum were distributed in each well to inoculate the microdilution plates. The inoculum was further diluted and 100 µL were spread twice on Sabouraud dextrose agar plates with a sterile Drigalski spatula. After 24–48 h of incubation at 35 °C, the colony forming units were counted to ensure the inoculum size and the viability of the conidia. The microplates were incubated at 35 °C under 95% humidity, and the minimal inhibitory concentrations (MICs) were determined spectrophotometrically at 48 h at a wavelength of 530 nm with the spectrometer MultiSkan FC (Thermo Fisher Scientific). All the experiments were run in duplicate.

### 2.5. Interpretation of the Results

The MICs alone and in combination were determined as the lowest concentrations that caused a complete inhibition as measured by a 90% of inhibition compared to the control according to spectrophotometric reading. For the calculation of the MIC_50_, the MIC_90_, and the geometric mean of isavuconazole, the MICs of all three sets were pooled together. For the calculation of the fractional inhibition concentration index (FICI), high off-scale MICs were converted to the next log2 dilutions. The FICI data were interpreted in the following way: FICI ≤ 0.5 = synergy, FICI > 0.5–4 = no interaction, and FICI > 4.0 = antagonism.

## 3. Results

For the combinations of isavuconazole with tacrolimus, cyclosporin A, or sirolimus tested against the 30 *Aspergillus* isolates by the checkerboard procedure, the MICs of the drugs alone, the MICs in combination, and the corresponding interaction for the lowest FIC indices are presented in Table 1. A summary of the results for all the combinations is presented in Table 2. The thirty isolates exhibited MICs for isavuconazole alone ranging from 0.25 to 16 µg/mL (Table 1) with a MIC_50_, MIC_90_, and geometric mean MIC of 1, 16, and 2.06 µg/mL, respectively. Isavuconazole MICs for *A. flavus*, *A. fumigatus*, *A. nidulans*, *A. niger*, and *A. terreus* ranged from 2 to 4, 1 to 16, 0.25 to 0.5, 4 to 16, and 0.5 to 1 µg/ml, respectively. Between experiments, the isavuconazole MICs were within +/− 1 log_2_ dilutions in 100% of the cases. Immunosuppressive drugs alone did not exhibit in vitro activity, except for four isolates. The tacrolimus MICs were >8 µg/mL, except for one *A. niger* (HEGP-6917) and two *A. terreus* (HEGP-6398, HEGP-6625) isolates. The cyclosporin A MICs were >8 µg/mL, except for one *A. niger* (HEGP-6917) and two *A. terreus* (HEGP-5599, HEGP-6398) isolates. For sirolimus, all the isolates exhibited MICs > 8 µg/mL. The interactions of isavuconazole with tacrolimus were synergistic for 50% of the isolates with FICIs ranging from 0.015 to 0.5. *A. fumigatus* isolates with mechanisms of resistance to azoles showed synergy for 60% of the isolates (three out of five isolates) and 100% synergy for all the *A. niger* isolates with isavuconazole MICs ≥ 8 µg/mL (four out of four isolates). For the other *A. niger* isolate (HEGP-6917) and two *A. terreus* isolates (HEGP-6398 and HEGP-6625), synergy was not detectable with the tacrolimus concentrations used on the microplates, because the MICs of tacrolimus alone were too low (0.25 µg/mL). Therefore, these isolates were excluded from the calculation of percentages of interaction of Table 2. For the combination of isavuconazole with cyclosporin A synergistic interactions were observed for 20% of the isolates. (FICI ranging from 0.13 to 0.5). Synergy was seen for 80% of the *A. niger* isolates; similar to what was observed for the combination with tacrolimus, synergy was obtained despite high MICs to isavuconazole of ≥8 µg/mL. For *A. terreus* synergy was seen for 40% of the isolates. For the combination of isavuconazole with sirolimus synergy was obtained for 10% of the isolates (FICI ranging from 0.19 to 0.38), comprising two *A. flavus* isolates and one *A. terreus* isolate. Antagonistic interactions were never observed for any combination tested.

## 4. Discussion

Immunosuppressive drugs such as calcineurin or mTOR pathway inhibitors are used as anti-rejection drugs in organ transplant, and allogeneic stem cell recipients. The calcineurin inhibitors lead to a reduced activity of cytokine genes, finally leading to the reduced proliferation of T lymphocytes [27,28]. Inhibitors of the mTOR pathway lead to an arrest of the cell-cycle in the late G1/S phase of T and B lymphocytes, preventing proliferation [29]. Beside these anti-proliferative properties, the drugs also possess intrinsic antifungal activity against yeasts [19,20] and filamentous [21,22] and dimorphic fungi [23]. Here, we found that immunosuppressors had no antifungal activity alone, except for four isolates (*A. niger* and *A. terreus*). It would be interesting to test a higher number of isolates to know if this is species or strain specific.

In vitro synergy between antifungals and immune suppressive drugs has been found for yeasts [30,31,32,33,34,35,36], and filamentous fungi such as the Mucorales [24,25,37,38], and *Aspergillus* species [39,40]. Nevertheless, in vitro indifference [21,41] and even antagonism has been reported for the combinations of voriconazole with tacrolimus or cyclosporin A against four *A. fumigatus* isolates and one *A. fumigatus* isolate, respectively [41]. Indifference has also been reported for the combinations of posaconazole or itraconazole with tacrolimus against *Aspergillus* biofilms [39]. One of the two studies demonstrating in vitro synergy between antifungals and immunosuppressors evaluated the combination of caspofungin in combination with tacrolimus, cyclosporin A, sirolimus, or other calcineurin inhibitors by a disc diffusion assay against 13 *Aspergillus*, mostly *A. fumigatus* isolates. The inhibition zones for tacrolimus or sirolimus in combination with caspofungin were significantly larger compared to those for caspofungin alone for the 10 *A. fumigatus* isolates at 48 h. The same results were seen for one *A. terreus* isolate, but for neither the other *A. terreus* isolate nor for the *A. flavus* isolate [40]. In this study, the immunosuppressive drugs showed poor in vitro activity when tested alone, in contrast with previous reports [21,42]. This could be related to differences in the technique used, and particularly, to the more stringent endpoint (90% inhibition) used in our study. The isavuconazole MICs of the tested *Aspergillus* isolates determined by ECUAST methodology were in the same range as previously reported [43]. The combination of isavuconazole with tacrolimus exhibited a synergistic effect (56% of the isolates) against *Aspergillus* species, including 60% of *A. fumigatus* isolates with mechanisms of resistance to azoles and all *A. niger* isolates with isavuconazole MICs ≥ 8 µg/mL. As tacrolimus is a known inhibitor of efflux pumps [44], it could be speculated that synergy may be more frequent in azole-resistant strains with an overexpression of efflux pumps. Therefore, it could be of interest to determine the level of expression of efflux pumps in our isolates. The presence of a known mechanism of resistance in our azole-resistant *A. fumigatus* strains (with *cyp51A* mutations and promotor alteration) did not rule out the possibility of higher efflux in these isolates. Our results are in accordance with a study that evaluated the interaction of voriconazole with tacrolimus against *Aspergillus* biofilms. The combination was tested against twenty *Aspergillus* biofilms and ten *A. fumigatus*, eight *A. flavus*, and two *A. terreus* isolates. Overall synergy was achieved for 60% of the tested isolates [39]. Why combinations of tacrolimus with isavuconazole or voriconazole exhibit synergy and combinations of tacrolimus with posaconazole or itraconazole exhibit only indifference remains unknown. It is possible that the different interactions are related to the steric structures of the molecules. Synergistic interactions between tacrolimus and isavuconazole may be of particular interest when tacrolimus analogs with lower immunosuppressive activity become available [45,46]. The combination of isavuconazole with cyclosporin A led to less synergistic interactions (20% of the isolates) than the combination with tacrolimus. The combination of isavuconazole with sirolimus was synergistic for 10% of the isolates. Similar results have already been seen for voriconazole in combination with the three immunosuppressors used in this study against *A. fumigatus*. All the interactions were indifferent [21].

It has to be pointed out that the concentrations for which synergistic interactions were achieved for tacrolimus, cyclosporin, and sirolimus in this study, were above the peak drug levels in clinical practice of 0.025 µg/mL, 1.2 µg/mL, and 0.02 µg/mL, respectively [47,48]. Nevertheless, analyses of the fractional inhibitory concentration indices are limited to the exploration of the MIC endpoints and the tested concentrations on the microplates. From these data, it cannot be excluded that synergistic interactions could be present at lower concentrations than those tested on the microplate. In another study, tacrolimus was tested by the same technique used in this study at subtherapeutic concentrations of 0.04–25 µg/mL in combination with amphotericin B or fluconazole. Synergy was obtained for 90 and 82% of the isolates, respectively. In the same study, the outcomes in solid organ transplant recipients with cryptococcosis receiving tacrolimus long-term therapy and amphotericin B or fluconazole were significantly better, regarding survival, than those of patients receiving only amphotericin B or fluconazole therapy without tacrolimus [49].

In summary, immunosuppressors can enhance the in vitro activity of isavuconazole against *Aspergillus* species. The best activity was seen for the combination of tacrolimus with isavuconazole. The combination was active against all the tested species, including *A. fumigatus* isolates with resistance to azoles and *A. niger* isolates with high isavuconazole MICs. The combination of cyclosporin with isavuconazole was active against all the *A. niger* isolates with high isavuconazole MICs. These in vitro results warrant further animal experiments.

## Figures and Tables

**Table 1:** Interaction of isavuconazole with tacrolimus, cyclosporin A, or sirolimus against *Aspergillus* species.

Species	Collection Number	MIC (µg/mL)					MIC (µg/mL)					MIC (µg/mL)				
		IVZ	TAC ^d^	IVZ/TAC	FICI	INTPN	IVZ	CYA ^d^	IVZ/CYA	FICI	INTPN	IVZ	SLM ^d^	IVZ/SLM	FICI	INTPN
*A. flavus*	HEGP-6097	4	16	2/1	0.5625	IND	4	16	4/0.12	1.0078	IND	4	16	1/2	0.375	SYN
*A. flavus*	HEGP-5899	4	16	0.5/8	0.625	IND	4	16	4/0.12	1.0078	IND	4	16	2/0.25	0.5156	IND
*A. flavus*	HEGP-4536	2	16	0.06/4	0.2813	SYN	2	16	2/0.12	1.0078	IND	2	16	1/1	0.5625	IND
*A. flavus*	HEGP-4251	2	16	0.03/4	0.2656	SYN	4	16	2/0.12	0.5078	IND	2	16	0.5/1	0.3125	SYN
*A. flavus*	HEGP-4114	2	16	0.5/4	0.5	SYN	2	16	2/0.25	1.0156	IND	2	16	1/1	0.5625	IND
*A. fumigatus*	HEGP-5780 ^b^	16	16	8/1	0.5625	IND	8	16	16/0.12	2.0078	IND	16	16	8/2	0.625	IND
*A. fumigatus*	HEGP-4020 ^c^	1	16	0.5/2	0.625	IND	2	16	1/0.12	0.5078	IND	1	16	0.5/2	0.625	IND
*A. fumigatus*	HEGP-4083 ^b^	16	16	4/4	0.5	SYN	16	16	16/0.12	1.0078	IND	16	16	16/0.12	1.0078	IND
*A. fumigatus*	HEGP-2659 ^b^	16	16	4/2	0.375	SYN	8	16	16/0.12	2.0078	IND	16	16	8/8	1.0	IND
*A. fumigatus*	HEGP-2664 ^b^	8	16	2/4	0.5	SYN	8	16	8/0.12	1.0078	IND	16	16	8/0.12	0.5078	IND
*A. fumigatus*	HEGP-R117	1	16	0.25/2	0.375	SYN	1	16	1/0.12	1.0078	IND	1	16	1/0.12	1.0078	IND
*A. fumigatus*	HEGP-R279	1	16	0.5/4	0.75	IND	1	16	1/0.12	1.0078	IND	1	16	0.5/0.12	0.75	IND
*A. fumigatus*	HEGP-R285	1	16	0.5/1	0.5625	IND	1	16	1/0.12	1.0078	IND	1	16	1/0.12	1.0078	IND
*A. fumigatus*	HEGP-R290	2	16	1/0.5	0.5313	IND	1	16	1/8	1.5	IND	2	16	1/8	1.0	IND
*A. fumigatus*	HEGP-R291	1	16	0.5/2	0.625	IND	1	16	1/0.12	1.0078	IND	1	16	1/0.12	1.0078	IND
*A. nidulans*	HEGP-5711	0.25	16	0.12/4	0.75	IND	0.5	16	0.25/2	0.625	IND	0.5	16	0.5/0.12	1.0078	IND
*A. nidulans*	HEGP-6169	0.5	16	0.12/4	0.5	SYN	0.5	16	0.25/2	0.625	IND	0.5	16	0.5/0.12	1.0078	IND
*A. nidulans*	HEGP-5492	0.5	16	0.25/1	0.5625	IND	0.5	16	0.25/2	0.625	IND	0.5	16	0.5/0.12	1.0078	IND
*A. nidulans*	HEGP-5521	0.5	16	0.25/0.5	0.5313	IND	0.5	16	0.25/4	0.75	IND	0.5	16	0.5/0.12	1.0078	IND
*A. nidulans*	HEGP-5329	0.5	16	0.25/1	0.5625	IND	0.5	16	0.25/2	0.625	IND	0.5	16	0.5/0.12	1.0078	IND
*A. niger*	HEGP-6071	16	16	0.25/0.25	0.0313	SYN	16	16	0.25/2	0.1406	SYN	16	16	16/0.12	1.0078	IND
*A. niger*	HEGP-6217	8	16	0.06/0.25	0.0234	SYN	8	16	1/2	0.25	SYN	8	16	4/2	0.625	IND
*A. niger*	HEGP-6475	16	16	0.06/0.25	0.0195	SYN	16	16	0.12/2	0.1328	SYN	16	16	8/0.12	0.5078	IND
*A. niger*	HEGP-6562	16	16	0.12/0.12	0.0156	SYN	16	16	0.25/2	0.1406	SYN	16	16	8/0.12	0.5078	IND
*A. niger*	HEGP-6917	4	0.25	0.03/0.25	1.0078	- ^a^	4	4	2/2	1.0	IND	8	16	4/0.12	0.5078	IND
*A. terreus*	HEGP-6625	0.5	0.25	0.12/0.12	0.75	- ^a^	1	16	0.06/2	0.1875	SYN	1	16	0.5/0.12	0.5078	IND
*A. terreus*	HEGP-6055	1	16	0.25/2	0.375	SYN	1	16	0.25/4	0.5	SYN	1	16	0.5/8	1.0	IND
*A. terreus*	HEGP-5599	0.5	16	0.03/0.12	0.0703	SYN	0.5	1	0.25/0.5	1.0	IND	0.25	16	0.12/8	1.0	IND
*A. terreus*	HEGP-5169	0.5	16	0.12/0.5	0.2813	SYN	0.5	16	0.25/2	0.625	IND	0.5	16	0.25/0.5	0.5313	IND
*A. terreus*	HEGP-6398	0.5	0.25	0.03/0.12	0.5625	- ^a^	0.5	1	0.06/0.5	0.625	IND	0.5	16	0.06/1	0.1875	SYN

MIC, minimal inhibitory concentration; FICI, fractional inhibitory concentration index; INTPN, interpretation; SYN, synergy (FICI ≤ 0.5); IND, no interaction (0.5 < FICI ≤ 4), ANT, antagonism (FICI > 4). IVZ, isavuconazole; TAC, tacrolimus; CYA, cyclosporin A; SLM, sirolimus; HEGP, Hôpital Européen Georges-Pompidou; ^a^ differentiation between synergy and no interaction not possible, as MICs of immune suppressors alone were too low (isolate excluded); ^b^ isolate with TR34/L98H alteration; ^c^ isolate with G54W mutation; ^d^ MICs for immunosuppressors reported as 16 μg/mL were >8 μg/mL.

**Table 2:** Summary of interactions of isavuconazole with tacrolimus, cyclosporin A, or sirolimus against *Aspergillus* species interpreted based on the fractional inhibitory concentration index.

Species (Number of Isolates)	% of Isolates with the Following Interaction ^a^								
	Synergy			No Interaction			Antagonism		
	TAC	CYA	SLM	TAC	CYA	SLM	TAC	CYA	SLM
*A. flavus* (5)	60	0	40	40	100	60	0	0	0
*A. fumigatus* (10)	40	0	0	60	100	100	0	0	0
*A. nidulans* (5)	20	0	0	80	100	100	0	0	0
*A. niger* (5)	100 ^b^	80	0	0	20	100	0	0	0
*A. terreus* (5)	100 ^b^	40	20	0	60	80	0	0	0
All (30)	56 ^b^	20	10	44 ^b^	80	90	0	0	0

^a^ all immunosuppressive agents were combined with isavuconazole; TAC, tacrolimus; CYA, cyclosporin A; SLM, sirolimus; ^b^ for the calculation of percentages 1 *A. niger* and 2 *A. terreus* isolates were excluded, as differentiation between synergy and no interaction was not possible with the concentrations of tacrolimus chosen on the plates.

## Congress Presentations

The results of this work (abstract number P059) were presented at the 9th Trends in Medical Mycology, 11th–14th October 2019 in Nice, France.
